# The adult growth hormone multicentric retrospective observational study: a 24-month Italian experience of adherence monitoring via Easypod™ of recombinant growth hormone treatment in adult GH deficiency

**DOI:** 10.3389/fendo.2023.1298775

**Published:** 2023-11-09

**Authors:** Antonio Mancini, Edoardo Vergani, Carmine Bruno, Claudia Giavoli, Matteo Spaziani, Andrea M. Isidori, Maura Arosio, Alfredo Pontecorvi

**Affiliations:** ^1^ Dipartimento di Medicina e Chirurgia Traslazionale, Unità di Endocrinologia e Diabetologia, Fondazione Policlinico Universitario A. Gemelli Istutito di Ricovero e Cura a Carattere Scientifico (IRCCS) – Università Cattolica del Sacro Cuore, Roma, Italy; ^2^ Endocrinology Unit, Fondazione Istutito di Ricovero e Cura a Carattere Scientifico (IRCCS) Cà Granda, Ospedale Maggiore Policlinico, Milan, Italy; ^3^ Department of Clinical Sciences and Community Health, University of Milan, Milan, Italy; ^4^ Department of Experimental Medicine, Sapienza University of Rome, Centre for Rare Diseases (Endo-European Reference Network on Rare Endocrine Conditions accredited), Policlinico Umberto I, Rome, Italy

**Keywords:** growth hormone, IGF-1, adherence, compliance, e-health, personalized medicine

## Abstract

**Introduction:**

Non-compliance to recombinant human growth hormone (rhGH) treatment is universally recognized as a key detrimental factor to achieve the expected clinical outcomes in adult GH deficiency (aGHD). The Easypod™ electronic device allows objective measurement of adherence. Adherence to treatment has been reported to be related with IGF-1 levels and consequently with clinical satisfactory results. The aim of this multicentric, observational, retrospective, 24- month study, is to objectively assess aGHD patients’ compliance to rhGH, using the Easypod™ device. Additionally, the study aims to compare the biochemical responses of adherent vs non-adherent patients.

**Methods:**

Forty-three patients (28 females and 15 males) affected by aGHD and equipped with Easypod™ from 3 Italian centers were included in the study. Adherence to treatment was defined as the proportion of injections correctly administered during the observational period, out of the expected total number of injections. All patients were evaluated for IGF-1, glucose, insulin, HOMA and QUICKI index, total/LDL/HDL cholesterol and triglycerides.

**Results:**

Mean adherence rate was consistently under 85% across the 2-year observation period (73% at year 2). A trend toward significant difference in adherence was shown when comparing female and male patients (respectively 76% and 61%) after a 2-year period. Among the anamnestic features, the prescribed frequency of administration of rhGH and the number of administered therapies appeared to be the most relevant adherence-influencing factors. A strong direct correlation between IGF-1 z-score and adherence to rhGH therapy was detected in the whole population.

**Discussion:**

Compliance to rhGH therapy is still a major issue in aGHD treatment. Adherence relates to therapy efficacy in aGHD. The use of Easypod™ could be beneficial for physicians to better manage aGHD patients and to achieve improved better biochemical and clinical responses.

## 1 Introduction

Adult growth hormone deficiency (aGHD) is a chronic disorder caused by congenital or acquired diseases affecting the hypothalamus-pituitary region (1). Recombinant human Growth Hormone (rhGH) is universally recognized as a useful therapy aiming at enhancing bone mineral density, decreasing cardiovascular and metabolic risk in aGHD patients, having as ultimate target the amelioration of their quality of life (2, 3). In chronic diseases, adherence to the prescribed regimen is a key point to achieve clinical success (4). Recombinant hGH therapeutic regimen, consisting of daily subcutaneous injections extending over many years, places a relevant psychological and physical burden on the patient, making adherence challenging (5). Moreover, whereas in childhood GHD linear growth represents an objective result of rhGH therapy, in aGHD the clinical results can be elusive and prone to subjectivity. Consequently, non-compliance and non-persistence to rhGH are the most common causes of treatment failure (6, 7). Compliance is the extent to which a patient follows the clinical prescription or medical advice (8, 9). Persistence is considered the percentage of patients continuing to use therapy after a specific period (10).

Non-compliance to rhGH represents a common finding and has a negative impact on treatment efficacy, leading to a rise in healthcare costs (11). Improving compliance and persistence in GHD management is a primary issue. Different solutions have been proposed, such as the development of smart devices for rhGH administration, the transmission of adherence data to digital cloud platforms (12) and the production of long-acting GH formulations (13). Adherence evaluation experienced a significant evolution during the last decades, following the implementation of digital solutions in medicine (e-Health). Twenty years ago, non-digital methods (where patients utilized a pen device for treatment administration and a paper diary to document their adherence) were the only available methodology. Later on, partially digital alternative emerged, with patients employing a pen device for treatment administration, and using a digital diary integrated into a mobile app or website to record their adherence. However, this methodology did not guarantee complete objectivity. Recently, fully digital alternative such as Easypod™ and Aluetta^®^ Smartdot™ have appeared; these devices automatically register adherence data and transfer them to the growzen™ ecosystem (12). The ecosystem enables the healthcare practitioners to quickly and easily access to highly reliable adherence data. The rapid recognition of patients with poor adherence allows treatment plan adjustments and consequently a better management (14).

While several data on children affected by GHD have been collected (15–20), no multicentric data of aGHD adherence are currently reported in the literature. Therefore, the aim of the present multicentric observational study was to provide real-world adherence data collected via Easypod™ in a large cohort of aGHD patients, and ultimately to correlate adherence data with clinical outcomes in such patients.

## 2 Materials and methods

The adult growth hormone multicentric retrospective observational study (AGHROS) was an observational, multicentric, retrospective study conducted to assess, as primary objective, the adherence rate to rhGH treatment over a 2-year period in adult patients affected by GHD. The enrolled patients received rhGH therapy using the easypod™ Clinical Kit, a system comprising an electronic, automated injection device (easypod™), with a docking station for recording rhGH administration data to enable objective monitoring of actual drug usage. The secondary objective was to evaluate how adherence to rhGH could modify IGF-1 z-score across the 2-year follow-up period and, eventually, serum parameters related to glyco-lipid metabolism.

Three Italian centers were involved in the study: Fondazione Policlinico Gemelli, IRCCS, Rome (coordinator center), Fondazione IRCCS Cà Granda Ospedale Maggiore Policlinico, Milan, and the Policlinico Umberto I, Rome.

A total of 43 participants (28 females, 15 males) were included in this study. They were recruited from the endocrinology outpatients of the respective hospitals, after being given an explanation about the study’s objectives and nature. The study was conducted in accordance with the declaration of Helsinki, as revised in 2013. The study protocol was approved by the Ethical Committee of Policlinico Gemelli (protocol ID 4148) and subsequently approved by the respective ethical committees of Fondazione IRCCS Cà Granda Ospedale Maggiore Policlinico Milan (protocol ID 0019981), and Policlinico Umberto I, Rome.

GHD was diagnosed by a dynamic test using growth hormone-releasing hormone (GHRH) 50 µg i.v. + arginine (0,5 g/Kg), with a peak GH response < 11 µg/L for individuals with a BMI < 25 kg/m^2^, < 8 µg/L for those with a BMI between 25 and 30 kg/m^2^ and < 4 µg/L for individuals with a BMI> 30 kg/m^2^ (1). Patients were tested accordingly to current guidelines (1, 3) or following a strong clinical suspicion, as previously reported (21). Previously performed Magnetic Resonance Imaging (MRI) was consulted to establish the etiology of hormone deficiency; patients who displayed a normal cerebral appearance were diagnosed as idiopathic GHD. GHRH plus arginine stimulation tests were repeated twice in idiopathic aGHD to confirm the diagnosis.

Only patients over 18 years, with a diagnosis of GHD according to the previous criteria were included. These patients were under rhGH treatment (Saizen^®^, Merck KGaA, Darmstadt, Germany) equipped with easypod™ and had provided written consent. Exclusion criteria were corticosteroid treatment (except for topic, inhaled and oral hydrocortisone as replacement regimen), bone dysplasia, active malignancy, history of cranial hypertension or active cranial hypertension, decompensated type 1 or 2 diabetes mellitus, autoimmune diseases under immunosuppressive treatment and other diseases characterized by low insulin-like growth factor-1 (IGF-1) levels, such as liver disease, malabsorption and malnutrition.

The study duration for each recruited patient was 2 years.

A baseline visit was scheduled for each patient participating in the study. The outcome measurements for both the primary and the secondary endpoints were assessed in five different moments: V0 (0 months), V1 (6 months), V2 (12 months), V3 (18 months) and V4 (24 months), respectively.

Easypod™ devices were supplied by Merck SpA (as commonly done for Saizen^®^ therapy). A support service, provided as part of Merck SpA Patient Support Program, was guaranteed to the enrolled patients in order to train them in correct device usage and replacement procedures, and managing any device malfunctions that might occur during the study. Pursuant to the observational nature of the study, the administration of rhGH treatment followed routine clinical practices, independent of the patient’s participation in the study.

The adherence to treatment for each patient was determined based on the injections recorded by the device, and it was estimated as the proportion of correctly administered injections during the observational period out of the total expected number of injections.

The threshold rate for high adherence was set as ≥85%, while low adherence was defined as ≤ 56%. Intermediate adherence was considered between 56 and 85% (20).

Blood samples were collected after overnight fasting into pyrogen-free tubes containing heparin as an anticoagulant. The following parameters were determined for all patients:

- Basal determination of IGF-1.
- Basal measurements of metabolic parameters: glucose, total cholesterol, low-density lipoprotein (LDL), high-density lipoprotein (HDL) and triglycerides.

Homeostasis model of insulin resistance (HOMA-IR), an insulin resistance index, was calculated according to the formula: [fasting insulin (U/ml)] * [fasting glucose (mmol/l)]/405 (22). QUICKI index was calculated according to the following formula: 1/log [fasting insulin (μUI/ml)] + log [fasting glucose (mg/dl)] (23).

Plasma concentrations of glucose, total cholesterol, HDL-cholesterol and triglycerides were measured by enzymatic assays. The intra-and inter-assay coefficients of variation (CV) for total cholesterol and triglycerides were < 1.5% and < 2.5%, respectively. The intra-and inter-assay CV for HDL-cholesterol were < 2.5% and < 3.0%, respectively. LDL cholesterol was calculated by the Friedewald’s equation: LDL cholesterol = total cholesterol - (HDL cholesterol + triglycerides/5).

At the Policlinico Gemelli hospital, serum concentrations of insulin and IGF-1 were measured by immunochemiluminometric assays on a Roche Modular E170 analyzer (Roche Diagnostics, Indianapolis, IN, USA). The intra- and inter-assay CV for insulin and IGF-1 were < 5.0% and < 7.0%, respectively. Standard Deviation Score (SDS) of IGF-1 were calculated according to the kit’s reference values table.

At the Fondazione IRCCS Cà Granda Ospedale Maggiore Policlinico,IGF-I concentrations were measured by a chemiluminescent immunometric assay (Immulite 2000 IGF-I; Siemens Medical Solutions Diagnostics, Los Angeles, CA), with an intra- and interassay coefficient of variation of 2.9 and 7.4%, respectively and calibrated according to IS 02/254 standard.

At the Umberto I Policlinico, IGF1 levels were assessed by radioimmunoassay (DIAsource Immunoassay, Belgium). The limit of detection was 0.25 ng/mL; intra-assay and inter-assay CV was respectively 8.8 and 9.1% at 168 ng/mL.

IGF-1 SDS is kit-specific and thus calculated differently in the three hospitals involved in the study.

### 2.1 Statistical analysis

The statistical analysis was performed using GraphPad Prism 9. D’Agostino and Pearson test was performed to all data preliminarily to evaluate their distribution within the studied population. Continuous variables were expressed as mean ± standard deviation (SD). If a normal distribution of data was displayed, the results were analyzed by means of Student’s unpaired t-test to evaluate the differences between groups and Pearson coefficient for correlation analysis. On the other hand, if data did not show a normal distribution, Mann-Whitney test was performed to study the differences between groups and Spearman coefficient for correlation analysis. The level of significance was set at 0.05.

## 3 Results

A total of 43 patients (28 females and 15 males) were enrolled in this study from three different Italian hospitals. Mean ± SD age was 54.1 ± 10.42 years whereas mean ± SD BMI was 27.3 ± 6.2 kg/m^2^.

28% of the patients were naïve to rhGH treatment at the time of the enrollment, while 72% were not.

**Figure 1:**
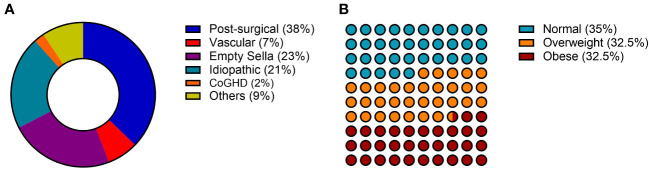
General characteristics of the AGHROS population. **(A)** Distribution of etiologies. **(B)** Graphic representation of aGHD patients according to BMI classes. BMI was categorized as follows: normal if between 18 and 25 kg/m^2^; overweight if between 25 and 30 kg/m^2^; obese if > 30 kg/m^2^.

The etiology of aGHD in our population was distributed as follows: 37% post-surgical hypopituitarism, 23% empty sella, 21% idiopathic, 7% vascular causes (Sheehan’s syndrome), 2% childhood onset GHD, and 9% other causes including non-functioning pituitary adenomas (NFPA) or pineal cysts (**Figure 1**).

The mean rhGH dose across the two-year follow-up period was 0.28 ± 0.1 mg/day for female patients and 0.25 ± 0.05 mg/day for male subjects. To date, 3 female patients were under estrogenic (and progestogen) therapies.

**Table 1** summarizes the general demographic and anamnestic features of the patients enrolled in the AGHROS study.

**Table 1:** Demographic and anamnestic description of the enrolled aGHD patients in the AGHROS study.

**Age (years)**	54.1 ± 10.42 (1.59)
**BMI (Kg/m^2^)**	27.3 ± 6.2
**Gender** Female Male	28 15
**Etiology** Post-surgical Vascular (Sheehan syndrome) Empty Sella Idiopathic CoGHD Others (Pineal Cyst/NFPA)	16 (37%) 3 (7%) 10 (23%) 9 (21%) 1 (2%) 4 (9%)
**Naïve to therapy** YES NO	12 (28%) 31 (72%)
**Mean rhGH dose (mg/day)** Female Male	0.28 ± 0.1 0.25 ± 0.05

**Figure 2:**
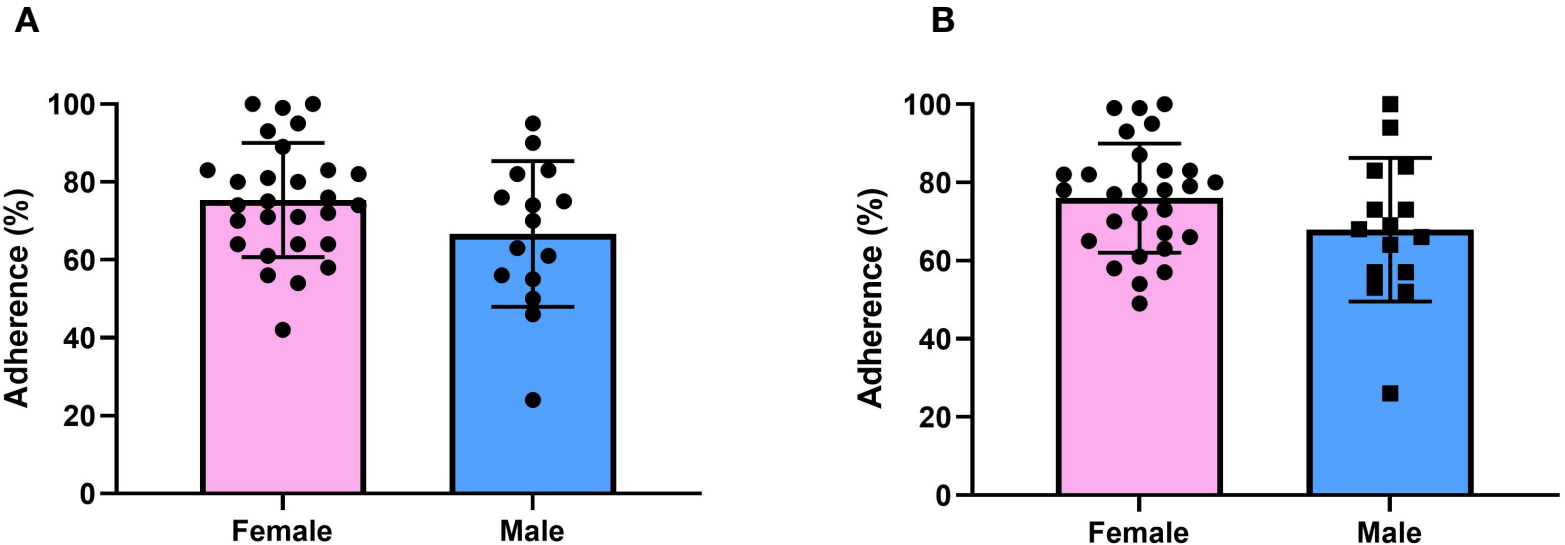
Adherence in female and male patients at 1-year and 2-year follow-up. **(A)** Adherence in different genders at 1 year. **(B)** Adherence in different genders at 2 years.

As shown in **Figure 2**, adherence to rhGH treatment was higher in female patients at the 1-year follow-up visit and after two years, although the differences were not statistically significant (p=0.08 and p =0.1).

No significant differences were observed in the mean ± SD body mass index (BMI), triglycerides, LDL and HDL levels at the beginning of the study, at one year and after two years within the whole population (**Table 2**). Similarly, when stratifying the population by gender, no significant differences were found, although a trend toward lower triglycerides was observed (p = 0.21). More importantly, due to the biological effect of rhGH, as commonly considered in the side effects evaluation of the molecule, no significant alterations in the glyco-metabolic profile were identified. Specifically, glycaemia, insulin levels and HOMA and QUICKI index did not change during the two-year assessment period.

**Table 2:** Metabolic profile of aGHD patients over the 2-year follow-up period.

	0		1 year follow-up		2 year follow-up	
	Mean	SD	Mean	SD	Mean	SD
Blood glucose (mg/dl) (32)	90.53	22.08	90.75	14.9	90.15	18.42
Insulin (mcU/ml) (16)	14.43	10.81	13.99	8.25	13.18	8.95
HOMA index (16)	3.36	2.81	3.31	0.61	3.20	2.60
QUICKI index (16)	0.35	0.06	0.34	0.04	0.34	0.04
Total Cholesterol (mg/dl) (35)	198.81	28.11	195.50	31.55	195.40	25.53
Triglycerides (mg/dl) (31)	149.42	68.69	120.14	43.64	128.06	46.48
HDL (mg/dl) (33)	54.50	14.29	57.05	20.01	55.37	15.07
cLDL (mg/dl) (31)	117.96	23.36	114.51	24.66	115.23	21.45
BMI (kg/m^2^) (43)	27.33	6.24	26.18	7.06	28.13	7.38
IGF-1 SDS (43)	-0.16	1.47	-0.13	1.36	0,07	1,36

The mean ± SD serum levels of glyco-lipid profile and BMI and IGF-1 z-score of the studied population are presented. The numbers in parentheses indicate the count of patients for whom complete data are available for the respective analyte.

When analyzing various potential anamnestic adherence-influencing features and performing a multiple regression analysis including variables like administration frequency, patients’ age, number of therapies administered, and whether patients were previously exposed to rhGH treatment or not, it was evident that both prescribed frequency administration (β= 0,1236; 95% C.I. 0,07780 - 0,1695 p=0.0001) and the number of therapies (β=0.01910; 95% C.I. -0,03517 - -0,003031; p=0.0211) were significantly associated with adherence over the two-year assessment period.

**Figure 3:**
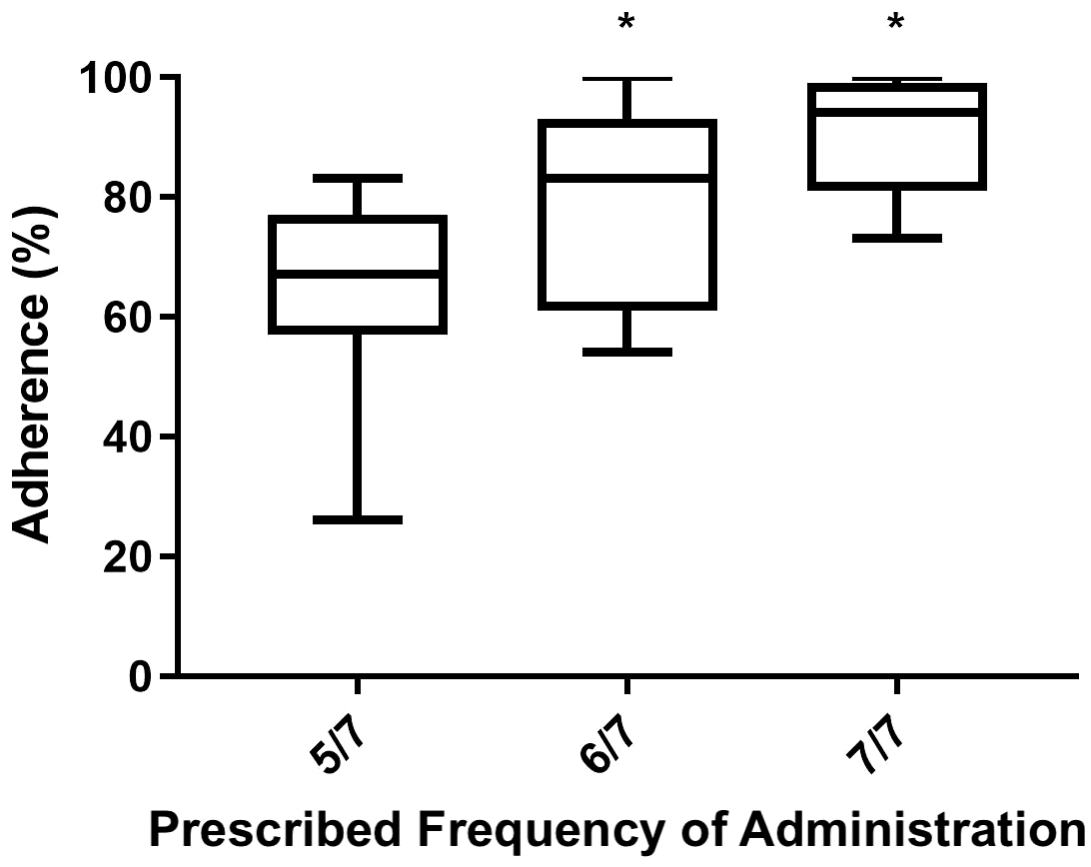
Box plot of Mean ± SD adherence to rhGH according to the prescribed frequency of administration. Patients following a rhGH administration schedule of 6 days a week and 7 out of 7 days showed higher adherence than those on 5 days a week schedule. *p < 0.05.

Between the two, the prescribed frequency of administration exhibited a higher power of association with the dependent variable (adherence to treatment). **Figure 3** shows by means of box plot how prescribed frequency administration affected adherence in the AGHROS population. Patients who adhered to a rhGH administration schedule of 6 days a week and 7 out of 7 days, showed greater adherence compared to those on a 5 days a week schedule.

**Figure 4:**
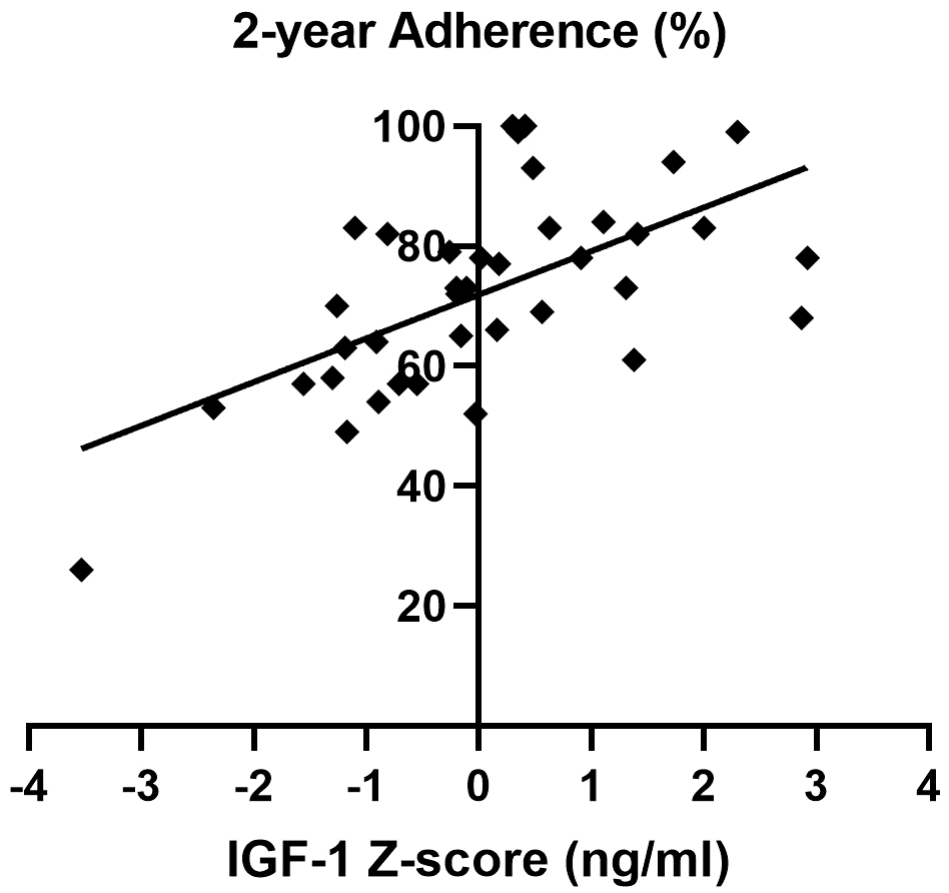
Correlation between adherence and IGF-1 z-score over the two-year follow-up of the AGHROS population. A direct correlation between adherence and IGF-1 z-score was shown (p< 0.05, Pearson r coefficient = 0.61).

Importantly, a significant and strong direct correlation between adherence and IGF-1 z-score during the two-year follow-up was detected (**Figure 4**).

**Figure 5:**
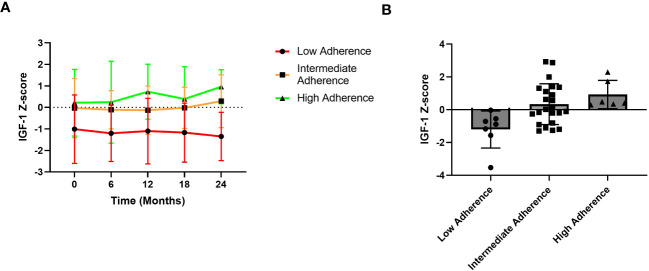
IGF-1 z-score trends over the years. **(A)** Trend graphic of Mean ± SD of IGF-1 z-score across the 2-year period. **(B)** IGF-1 Z-score distribution according to adherence groups. Patients with high adherence show a better IGF-1 z-score trend compared to those with low adherence.

Finally, **Figure 5** shows IGF-1 z-score trend over the 2-year follow-up period based on different adherence categories. In detail, the high adherence group consisted of patients with adherence ≥ 85%, the intermediate adherence group included those with adherence between 56 and 85% whereas the low adherence group comprised patients with adherence ≤ 56%.

## 4 Discussion

The AGHROS study is the first multicentric observational study focused on objectively assessing adherence to rhGH treatment in aGHD. The multicentric nature of the study is essential for gathering a larger number of patients and evaluating different hospital experiences in the management of aGHD. The population enrolled in the AGHROS study was distributed similarly to those enrolled in the ANSWER study, one of the biggest cohorts of aGHD patients ever assembled. In detail, within our cohort, 21% of patients suffered from idiopathic GHD, whereas in the ANSWER study the percentage was 24% (24, 25). In the AGHROS cohort, post-surgical aGHD was the most common (**Figure 1A**). Overall, females comprised the 65% of the AGHROS, again similarly to what depicted in the ANSWER study. Unlike the ANSWER study, our patients are equally distributed in normal weight, overweight and obese categories (**Figure 1B**). Mean GH dose was comparable to those estimated in the Nordinet^®^ IOS program (24, 25). In conclusion, the epidemiologic features of our cohort were partly like those described in the two biggest aGHD cohort study programs. Of interest, the 72% of our patients were non-naïve to treatment, whereas the 28% were naïve (**Table 1**). Keeping in mind this observation is useful to interpretate some of the clinical data here presented, such as the IGF-1 SDS and the lack of significant change in metabolic parameters during the two years of adherence evaluation (**Table 2**).

Our data confirmed low adherence in aGHD population on rhGH treatment (6). Mean adherence in the whole population was nearly 73% after two years of observation, far inferior to those of pediatric population collected by the ECOS studies (15–20). This could be explained given the different treatment results gained in pediatric versus aGHD: on one hand, height gain is an evident and gratifying result, on the other hand, IGF-1 correction and body composition modification could be more elusive and less visible. Furthermore, differently from our previous report, when evaluating adherence data according to the gender, males appeared to be less adherent (**Figure 2**), even though without statistical significance (6). The WHO reported 80% as the threshold to consider the patient compliant to a chronic therapy (26). Koledova et al. confirmed the indication in children with short stature treated with rhGH, as the value is related to better growth outcomes (18). Meanwhile, other studies chose 85% as the threshold for “good adherence”, and 56% as the threshold for inadequate adherence (20). As above stated, non-compliance is one of the most important causes of chronic treatment failure. The Easypod™ device and the related digital ecosystem could be useful to promptly identify patients with low adherence and to support timely treatment optimization (27, 28). The gathered data support the need for increased attention to male patients affected by aGHD. Furthermore, as previously demonstrated, male and females could present different reasons to non-compliance (29). Considering gender differences and identifying the underlying reasons could provide a novel approach to address and resolve compliance issues.

In addition to gender, various anamnestic features of our patients have been explored according to adherence data. By utilizing multiple regression analysis, it was found that the number of administered therapies and the prescribed frequency of administration significantly impact adherence to rhGH treatment. Between the two factors, the prescribed frequency of administration exhibited a stronger power of association. It is known that patients who take multiple medications are less adherent than patients who have lighter regimens (30–32). Moreover, some elements led us to hypothesize patients’ lack awareness regarding the significance and advantages of rhGH replacement therapy. Several patients from the AGHROS study were undergoing other endocrinological treatment, such as levothyroxine and hydrocortisone as replacement therapy due to impaired pituitary function. These patients are more susceptible to problems related to thyroid and adrenal deficiencies due to the potentially life-threatening consequences. Adrenal and thyroid replacement therapies are generally regarded as more crucial than quality of life and the proven benefits of rhGH. Finally, the oral formulation of thyroid and adrenal replacement therapy is more accepted than subcutaneous rhGH injections. In our opinion, despite a large literature on the morbidity and mortality related to aGHD. The clinical perception of aGHD is still limited, both by physicians and patients. Physicians’ awareness (and consequently communication skills) is essential, since patient’s knowledge about medication regimen is considered a strong predictor of adherence (33). Surprisingly, our patients showed better adherence to treatment when on six or seven days a week schedule, compared to five days a week one. It is presumable that the introduction of two days a week of therapy avoidance, could represent a confusion element for the patient, thus increasing forgetfulness, which is considered one of the main causes of unintentional non-adherence (34). Another possible hypothesis patient’s misperception of rhGH therapy: the avoidance of two days of therapy a week, in addition to the subtle clinical presentation of aGHD, may allow patients to think that rhGH could be skipped.

Unlike adherence, the persistence to rhGH was 100% in our study, although it was not an objective of the AGHROS, as longer period of evaluation is needed. However, several studies reported higher persistence with lighter treatment schedules. Consequently, to answer the problem of patients’ persistence and acceptance of chronic aGHD therapy, long acting GH have been developed (13). To date, no studies have questioned adherence to treatment as primary endpoint. The current evidence is that long-acting GH are non-inferior to daily GH when evaluating adherence to treatment (35). The REAL 3 is the only study which showed better compliance with long-acting GH than daily GH (36). No studies have ever compared adherence outcomes between long-acting GH and Saizen^®^, and its integrated digital ecosystem. Finally, no differences were detected between idiopathic aGHD and “organic” ones, nor between naïve and non-naïve patients.

Only few studies focused on adherence in aGHD and the reasons associated to patients’ non-compliance. Kreitschmann-Andermahr et al. collected patients’ perception about rhGH treatment by means of three *ad hoc* questionnaires: older age was significantly associated with better adherence to rhGH, while injection side effects, duration of treatment or device used, were not. Fear of side effects, dislike of injections and lack of belief in treatment, represents the most common causes of rhGH refusal (37). As previously stated, by means of objective acquisition of adherence data, in our cohort we showed that the number of administered therapies and the prescribed frequency of administration, significantly impacted on treatment adherence.

An intuitive, yet fundamental, result of the AGHROS study, is the strong direct correlation between IGF-1 SDS and adherence to treatment. The more the patients are adherent to rhGH, the better is the IGF-1 SDS. Before checking the IGF-1 on outpatient visit, adherence should be considered. This could also lead to rhGH escalation, especially in those patients with high adherence and IGF-1 SDS >2. IGF-1 is universally considered the sole and highly reliable target of rhGH therapy, since higher IGF-1 levels, in treatment range, are associated to better outcome on metabolic parameters, body weight composition and cardiovascular features (38–40). According to Le Corvoisier et al. meta-analysis, IGF-1 concentrations correlate with rhGH therapy efficacy. An increase in IGF-1 >89% is associated to a significant improvement in heart function and reserve, allowing to classify patients in responders or non-responders (41). We suggest adding an objective evaluation of adherence before considering whether a patient is a responder or not.

The novelty of the study, the multicentric nature, the two-year follow-up, the use of Easypod™ (consequently the reliability of adherence data) and the relatively “wide” cohort according to the rarity of the disease, represent the main strength of the AGHROS study. However, some limitations and restrictions must be considered. The AGHROS study is observational and retrospective, and this design does not allow to find any cause-effect relationships, for which larger population studies and different designs are required. Clinical parameters modifications, such as metabolic assessment and IGF-1 SDS before and after rhGH, did not represent the primary objective of the AGHROS study. As such, we accepted both naïve and non-naïve patients. Moreover, body composition measures have not been considered. In case of future evaluations on adherence impact on clinically significant aGHD impairments, it could be useful to enroll only patients naïve to treatment. Finally, HOMA-IR and QUICKI are commonly used as indices of insulin resistance/sensitivity in large epidemiological studies, and their adoption in a small population might be less indicated.

Compliance to rhGH therapy is still a major issue in aGHD treatment. Adherence relates to therapy efficacy in aGHD. During the last years several efforts have been made to empower patients’ compliance: new devices, long acting rhGH and e-health applications. In conclusion, the use of Easypod™ and the connected digital ecosystem could be beneficial for physicians to better manage aGHD patients and to gain better biochemical and clinical responses.

## Data availability statement

The raw data supporting the conclusions of this article will be made available by the authors, without undue reservation.

## Ethics statement

The studies involving humans were approved by Ethical Committee of Fondazione Policlinico Gemelli, IRCCS (protocol ID 4148). The studies were conducted in accordance with the local legislation and institutional requirements. The participants provided their written informed consent to participate in this study.

## Author contributions

AM: Conceptualization, Methodology, Project administration, Writing – review & editing. EV: Conceptualization, Data curation, Formal Analysis, Investigation, Methodology, Project administration, Writing – original draft. CB: Data curation, Formal Analysis, Investigation, Writing – review & editing. CG: Investigation, Writing – review & editing. MS: Investigation, Writing – review & editing. AI: Supervision, Writing – review & editing. MA: Supervision, Writing – review & editing. AP: Project administration, Supervision, Writing – review & editing.
